# Functional Outcomes of Extra-Articular Distal Humerus Fractures Managed With Anatomical Locking Plates: A Prospective Cohort Study

**DOI:** 10.7759/cureus.92064

**Published:** 2025-09-11

**Authors:** M Shamanth, K S Vineeth, R Kiran, Harish Karigowda

**Affiliations:** 1 Orthopaedics, Adichunchanagiri Institute of Medical Sciences, Mandya, IND

**Keywords:** anatomical lcp, extra-articular distal humerus fracture, mayo elbow performance score, paratricipital approach, single-column fixation

## Abstract

Introduction

Extra-articular distal humerus fractures account for approximately 16% of humerus fractures and 3% of adult fractures. These injuries present treatment challenges due to their periarticular location, frequent comminution, and often osteoporotic bone quality in elderly patients. Current management remains controversial between conservative immobilization and surgical fixation. The purpose of this study was to evaluate the functional outcomes of extra-articular distal end humerus fractures treated with open reduction and internal fixation (ORIF) with anatomical locking plates via the paratricipital approach.

Methods

We conducted a prospective study of 30 adults with AO Foundation/Orthopaedic Trauma Association (AO/OTA) type 13-A2/A3 fractures treated at our institution between October 2022 and October 2024. All patients underwent ORIF using anatomical locking plates via the paratricipital approach. Outcomes were assessed using the Mayo Elbow Performance Score (MEPS), range of motion measurements, and radiographic evaluation over 12 months of follow-up. Ethical clearance and informed consent were obtained for all procedures.

Results

At the 1-year follow-up, 76.6% of patients achieved good-to-excellent MEPS scores, with a mean score of 81. The MEPS showed statistically significant improvement from a mean of 61.3 at one month to 81.0 at 12 months (p<0.001). The mean elbow flexion was 110.8°, with an extension deficit of 8.2°, and a mean supination/pronation of 67.1°. All fractures achieved radiographic union by an average of 12.4 weeks. Complications included transient radial nerve palsy in two patients (6.7%), superficial infection in one patient (3.3%), and implant failure in one patient (3.3%).

Conclusion

ORIF using anatomical LCP via the paratricipital approach provides stable fixation, facilitates early mobilization, with excellent functional outcomes for extra-articular distal humerus fractures, with minimal complications. This technique is particularly advantageous for preserving the radial nerve and addressing osteoporotic bone. Further comparative studies with longer follow-up are recommended to validate these findings.

## Introduction

Distal humerus fractures account for approximately 16% of all humeral fractures and 3% of fractures in adults, presenting a significant clinical challenge due to their periarticular location, comminution, and the often osteoporotic bone quality in elderly patients [1,2]. These fractures typically exhibit a bimodal distribution, with high-energy trauma (e.g., road traffic accidents) affecting younger individuals and low-energy falls predominating in older populations [3]. Extra-articular distal humerus fractures, classified as AO Foundation/Orthopaedic Trauma Association (AO/OTA) type 13-A, require meticulous management to restore function and alignment while minimizing complications such as stiffness, non-union, and nerve injuries [4].

Open reduction and internal fixation (ORIF) is the current standard for displaced or unstable distal humerus fractures, while non-operative care is reserved for stable, minimally displaced fractures or patients unfit for surgery. Although non-operative management (e.g., functional bracing) is associated with malalignment and non-union rates of up to 24%, surgical fixation, particularly ORIF with locking compression plates (LCPs), has emerged as the gold standard for displaced fractures [5,6]. Anatomically contoured LCPs offer biomechanical advantages, including angular stability and improved screw purchase in the distal fragment, enabling early mobilization and better functional outcomes [7,8]. The paratricipital approach further enhances surgical outcomes by preserving the extensor mechanism and minimizing soft tissue dissection, thereby reducing complications like radial nerve palsy and infection [9,10].

In this study, 30 patients with extraarticular distal humerus fractures were evaluated for their functional and radiological outcomes (AO type 13-A2/A3) treated with ORIF using anatomical LCPs via a paratricipital approach. We evaluate the effectiveness of this technique in achieving fracture union, restoring elbow function (measured by the Mayo Elbow Performance Score, MEPS), and minimizing complications.

## Materials and methods

Study design

This was a prospective study conducted at Adichunchanagiri Institute of Medical Sciences, B G Nagara, from October 2022 to October 2024 after obtaining Institutional Ethics Committee approval (IEC No: AIMS/IEC/74/2022). Throughout the study, all participants gave their written informed consent.

Study population

Thirty consecutive adult patients aged 18 to 60 years who presented with closed extra-articular distal humerus fractures classified as AO/OTA types 13-A2 or 13-A3 participated in the study. The study included skeletally mature patients within the specified age range, with closed, extra-articular distal humerus fractures, and no history of prior elbow surgery or pre-existing deformities on the ipsilateral side. Intraarticular extension of fractures (AO/OTA 13-B or 13-C), open fractures of any Gustilo-Anderson grade (I to III), pathological fractures, and neurological injuries, or if they were polytrauma patients with an Injury Severity Score (ISS) greater than 16, were excluded.

Procedure

All participants underwent a comprehensive preoperative evaluation, including a detailed clinical examination with documentation of neurovascular status, standard anteroposterior and lateral radiographs of the elbow, and CT scans when articular involvement was suspected. Routine haematological and biochemical investigations were performed for preoperative optimization.

All surgical procedures were performed by a single senior orthopedic surgeon following a standardized protocol. Patients were positioned in lateral decubitus with the affected arm placed over a padded bolster, and a non-sterile tourniquet was applied. A posterior midline incision approximately 10-12 cm in length was made, followed by a paratricipital (triceps-sparing) approach. This involved developing medial and lateral windows between the respective intermuscular septa and triceps muscle, with careful identification and protection of the radial nerve throughout the procedure. The fracture was reduced under direct visualization using pointed reduction clamps. The fracture was fixed using extra-articular locking compression plates of 3.5 mm, which were contoured to match the posterolateral column anatomy. A minimum of six cortices of fixation were obtained, both proximal and distal to the fracture site. Intraoperative fluoroscopy was used to confirm satisfactory reduction and appropriate hardware positioning.

Postoperatively, patients were immobilized in an above-elbow slab with the elbow at 90° flexion for two weeks. Active finger movements were encouraged from the first postoperative day. A supervised, progressive rehabilitation protocol was implemented, beginning with active-assisted range of motion exercises during the initial 0-2 weeks, followed by progressive strengthening from 2-6 weeks, and finally allowing gradual return to functional use as tolerated after 6 weeks.

Follow-up assessments were conducted at standardized intervals: two weeks for suture removal, six weeks later, three months later, six months later, and one year after surgery. The primary outcome measures included functional assessments using MEPS and radiological evaluation of fracture union, defined as bridging calluses visible in three of four cortices on orthogonal radiographs. Secondary outcomes included range of motion measurements using a standard goniometer and documentation of any complications such as infection, nerve injury, or hardware failure.

Data collection and analysis 

In this study, statistical analysis was performed using SPSS version 26 (IBM Corp., Armonk, NY, US). Continuous variables are expressed as mean + standard deviation, while categorical variables are expressed as percentages. A repeated measures analysis of variance (ANOVA) was used to analyze changes in MEPS scores across time points, with a p-value of 0.05 considered statistically significant.

## Results

The study population consisted of 30 patients whose mean age was 40.1 years (range 19-60 years), with 20 males (66.7%) compared to 10 females (33.3%). Fracture distribution between sides was nearly equal, with 16 cases (53.3%) involving the left humerus and 14 cases (46.7%) affecting the right side. Most injuries were the result of road traffic accidents, accounting for 17 cases (56.7%), while the remaining 13 cases (43.3%) were the result of simple falls. According to the AO/OTA classification system, 19 fractures (63.3%) were classified as type A2 (simple metaphyseal) and 11 fractures (36.7%) as type A3 (multifragmentary metaphyseal), as shown in Table 1.

**Table 1:** Demographic and clinical characteristics of the study population

Variable	Categories	Number of patients, n (%)
Age group (years)	18–30	9 (30%)
	31–40	9 (30%)
	41-50	5(16.7%)
	>51	7(23.3%)
Gender	Male	20 (66.7%)
	Female	10 (33.3%)
Mechanism of injury	Road traffic accident	17 (56.7%)
	Fall from height	13 (43.3%)
Side of fracture	Right	14 (46.7%)
	Left	16 (53.3%)
Fracture pattern	A2 (Simple)	19 (63.3%)
	A3 (Multifragmentary)	11 (36.7%)

Functional outcomes demonstrated progressive improvement throughout the follow-up period. The final 12-month assessment showed a mean flexion of 110.8 degrees with a standard deviation of 22.5 degrees, ranging from 70 to 140 degrees. From full extension to a 30-degree deficit, the mean extension deficit was 8.2 degrees with a standard deviation of 9.1 degrees. The mean total arc of motion measured 102.6 degrees with a standard deviation of 23.1 degrees. Pronation and supination were measured at 67.1 degrees and 7.9 degrees, respectively, with no significant changes.

**Figure 1:**
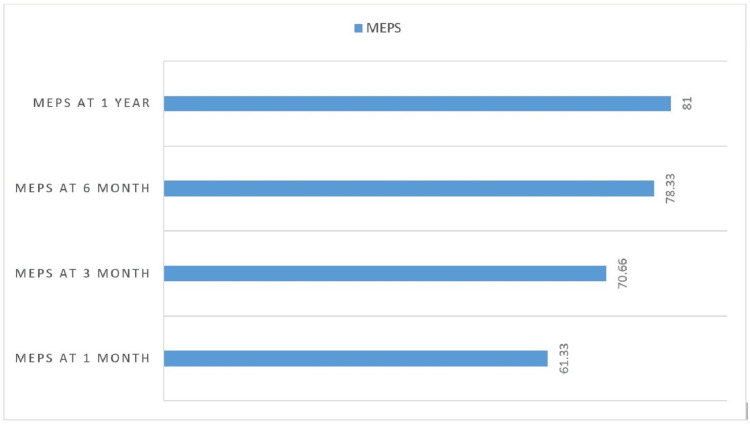
Comparing MEPS between time intervals MEPS: Mayo Elbow Performance Score

There was a significant improvement in MEPS scores during the follow-up period. The final one-year assessment revealed 33.3% (n=10) with excellent outcomes, 43.3% (n=13) with good results, 16.7% (n=5) with fair outcomes, and only 6.7% (n=2) remaining in the poor category, as mentioned in Table 2. Statistical analysis confirmed significant improvement in mean MEPS scores from 61.3±7.9 at one month to 81.0±11.5 at one year, as illustrated in Figure 1.

**Table 2:** Functional outcomes using the MEPS score at defined postoperative intervals (n=30) *p-values are based on repeated measures ANOVA test; p-value of ≤0.05 showed statistically significant difference in MEPS scores across time points MEPS: Mayo Elbow Performance Score; ANOVA: analysis of variance

MEPS Grade	1 month, n (%)	3 months, n (%)	6 months, n (%)	1 year, n (%)	p value
Excellent	0	1 (3.3%)	5 (16.6%)	10 (33.3%)	0.0001^*^
Good	0	16 (53.3%)	15 (50.0%)	13 (43.3%)	
Fair	23 (76.6%)	8 (26.6%)	7 (23.3%)	5 (16.6%)	
Poor	7 (23.3%)	5 (16.6%)	3 (10.0%)	2 (6.6%)	

**Figure 2:**
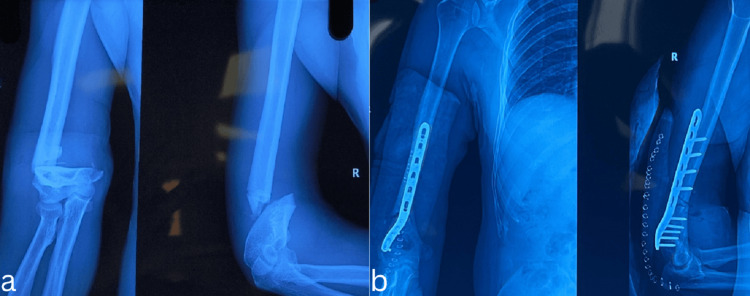
A case of A2 extra-articular distal humerus fracture (a) preoperative anteroposterior and lateral radiograph of an extra-articular fracture of the distal humerus, (b) immediate postoperative radiograph showing adequate reduction with the use of anatomical plating

**Figure 3:**
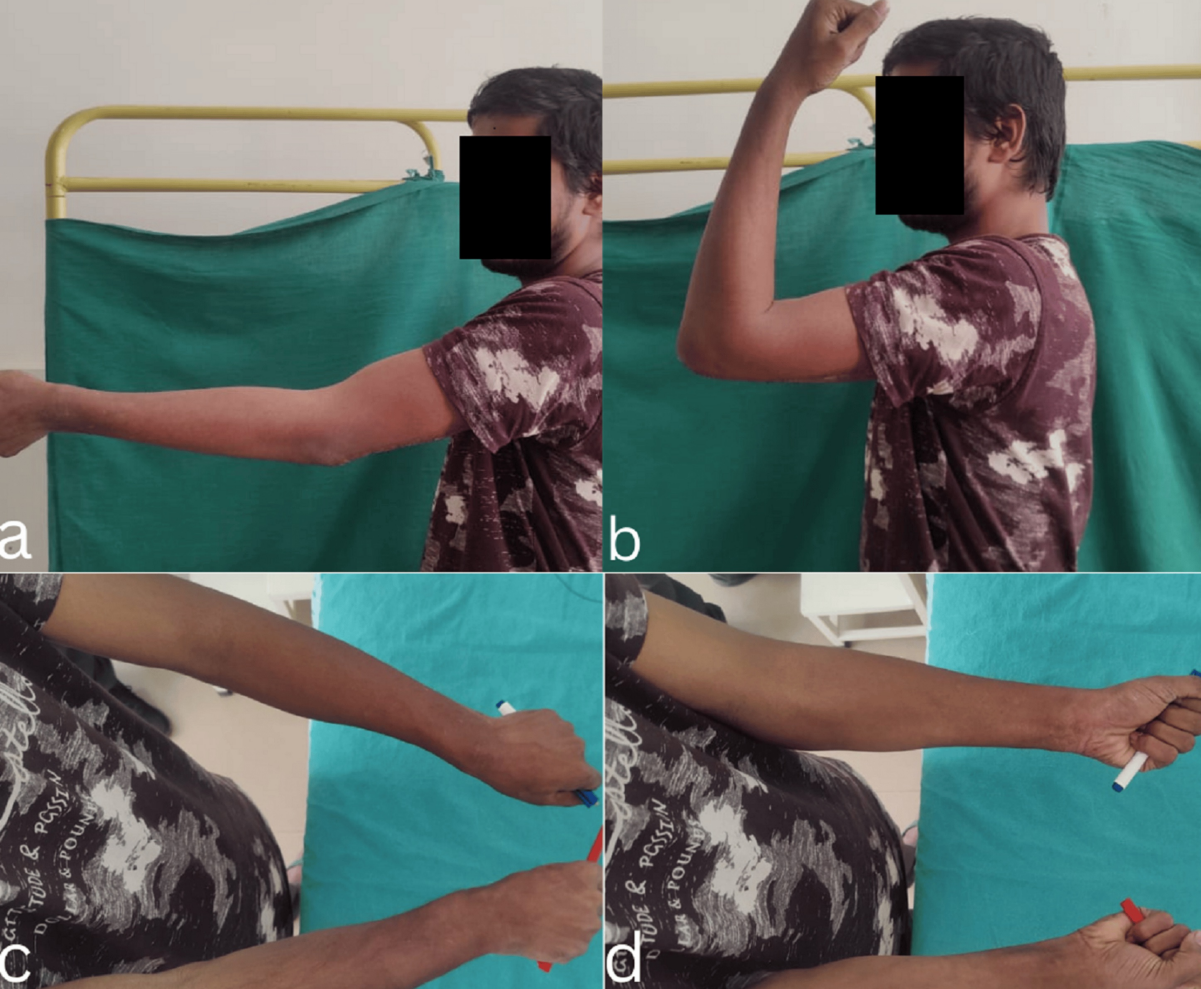
Clinical picture showing a good range of movements of the elbow joint at the final follow-up (a) extension, (b) flexion, (c) pronation, (d) supination

Radiological outcomes were consistently positive, with all fractures achieving union by six months postoperatively. In this study, the mean time to radiological union was 12.3 weeks, with a standard deviation of 2.1 weeks. Healing followed a predictable pattern, with early callus formation visible at 6-8 weeks, bridging callus across at least three cortices by 12 weeks, and complete remodelling observed in all cases by six months. Radiographs and clinical images are presented below (Figures 2, 3).

**Figure 4:**
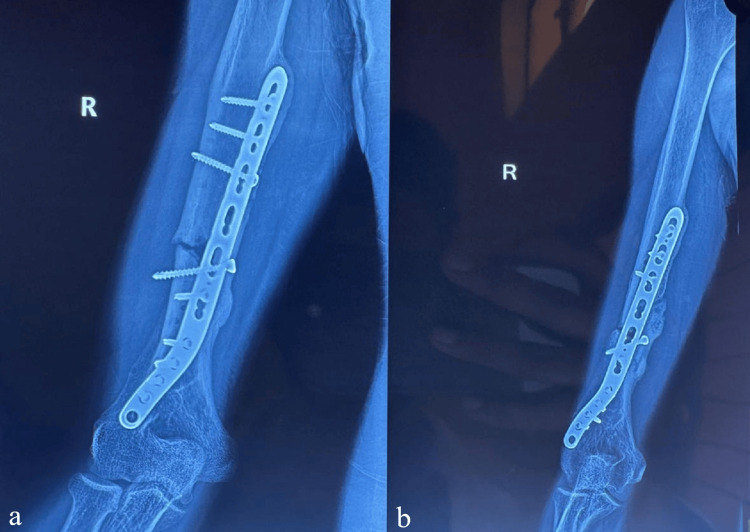
Case of implant failure at eight weeks (a): Anteroposterior (AP) X-ray of the distal humerus; (b): lateral X-ray of the distal humerus

Complications included 2 cases (6.7%) of radial nerve neuropraxia, both resolving completely by 3 months with conservative management, and 1 case (3.3%) of superficial infection, which was treated with oral antibiotics. In 3 patients (10%), elbow stiffness (flexion<100°) was diagnosed and improved after physiotherapy. One patient (3.3%) experienced implant failure at eight weeks due to premature weight-bearing, illustrated in Figure 4, requiring revision fixation with a longer plate and bone grafting, which subsequently healed uneventfully. No cases of heterotopic ossification or hardware irritation requiring removal were noted.

Functional recovery timelines were encouraging. At a mean of 8.2 weeks, patients were able to carry out daily activities. Light work resumed at 10.1 weeks, with a standard deviation of 2.3 weeks. Among the 18 patients engaged in heavy labor occupations, all were able to return to their previous level of work by 6 months postoperatively. These results collectively demonstrate that anatomical locking plate fixation through the paratricipital approach provides reliable fracture stabilization, facilitates satisfactory functional recovery, and maintains low complication rates in the management of extra-articular distal humerus fractures. In combination with a structured rehabilitation program, this surgical technique showed statistically significant improvements in MEPS scores over the follow-up period of one year.

## Discussion

This prospective cohort demonstrates that ORIF with anatomical locking plates via a paratricipital approach achieved reliable union and favorable early functional outcomes in adults with extra-articular distal humerus fractures, with significant improvement in MEPS scores from 61.3 at 1 month to 81.0 at final follow-up (p<0.001). These findings compare well with previous studies reporting mean MEPS ranging from 78 to 89 after similar fixation methods [3,5,6]. The preservation of forearm rotation (mean 67.1° pronation/supination) and achievement of functional elbow motion (mean arc 102.6°) underscore the advantages of this surgical technique. Our functional outcomes were consistent with prior reports of single-column fixation. Jain et al. reported MEPS of 84.2, slightly higher than our 81.0, likely reflecting exclusion of multifragmentary fractures [3]. Similarly, Kumar et al. reported union at 12.8 weeks, aligning with our 12.3 weeks [7]. The functional arc of motion (mean 102.6°) in our study exceeds the 92° reported by Trikha et al. in their 36-patient series, potentially reflecting our earlier initiation of motion therapy at 2 weeks postoperatively.

The 100% union rate observed in our series supports the biomechanical stability provided by modern locking plate technology. Our mean time for union was 12.3 weeks, which aligns with Kumar et al.'s report of 12.8 weeks [7], though slightly longer than Jain et al.'s 10.5 weeks [3]. This difference may reflect our inclusion of more complex A3 fractures (36.7% of cases). The single implant failure (3.3%) occurred in an active laborer who prematurely returned to heavy lifting, emphasizing the need for strict postoperative activity restrictions, particularly in osteoporotic bone.

While our complication rate (16.7%) appears lower than some dual-plate series, direct comparisons cannot be made due to the absence of a control group. The absence of ulnar neuropathy and heterotopic ossification in this series may reflect the nerve-sparing and soft-tissue preserving aspects of the paratricipital approach [11]. The radial nerve palsy rate in our study (6.7%) was lower than the 14-16% reported with extensive dual-plating approaches [12], supporting the nerve-sparing benefits of the paratricipital approach. None of the patients developed heterotrophic ossification, which is one of the known complications of elbow trauma [13]. The posterolateral plate position avoided the olecranon impingement reported in 11% of cases by Korner et al. with medial plating [14]. The paratricipital approach's soft-tissue preservation likely contributed to our lower stiffness rate (10%) versus the 15-20% reported with triceps-reflecting approaches [15].

Our findings support the feasibility and safety of paratricipital single-column fixation for extra-articular distal humerus fractures, achieving predictable union and satisfactory early outcomes. However, a definitive assessment of comparative effectiveness and long-term durability requires larger controlled studies with extended follow-up.

Limitations

This study has several limitations. First, the single-arm design without a comparator precludes conclusions on relative effectiveness. Second, the modest sample size limits statistical power. Third, elderly, open, and pathological fractures were excluded, restricting generalizability. Fourth, rehabilitation adherence was not formally quantified. Finally, follow-up was limited to 12 months, insufficient to assess long-term complications such as post-traumatic arthritis or late hardware failure.

## Conclusions

This prospective study demonstrated that anatomical locking compression plate fixation via a paratricipital approach achieved reliable union and favorable functional outcomes in extra-articular distal humerus fractures (AO/OTA 13-A). Over one year of follow-up, Mayo Elbow Performance Scores improved significantly (61.3 to 81.0, p<0.001), with preserved forearm rotation and a 100% union rate. Complications were infrequent, with only one implant failure (3.3%) and two transient radial nerve palsies (6.7%). The paratricipital approach provided adequate stability, even in multifragmentary (A3) fractures, while minimizing soft tissue disruption and preserving the extensor mechanism. These features contribute to early return to function and a low incidence of stiffness.

Overall, our findings support the feasibility and safety of paratricipital single-column fixation for extra-articular distal humerus fractures. However, confirmation of comparative effectiveness and long-term durability requires larger controlled trials with extended follow-up.
